# Organisation and dynamics of individual DNA segments in topologically complex genomes

**DOI:** 10.1093/nar/gkaf925

**Published:** 2025-11-11

**Authors:** Saminathan Ramakrishnan, Guglielmo Grillo, Auro Varat Patnaik, Luca Tubiana, Davide Michieletto

**Affiliations:** School of Physics and Astronomy, University of Edinburgh, Peter Guthrie Tait Road, Edinburgh EH9 3FD, UK; Physics Department, University of Trento, via Sommarive 14, I-38123 Trento, Italy; INFN-TIFPA, Trento Institute for Fundamental Physics and Applications, I-38123 Trento, Italy; School of Physics and Astronomy, University of Edinburgh, Peter Guthrie Tait Road, Edinburgh EH9 3FD, UK; Physics Department, University of Trento, via Sommarive 14, I-38123 Trento, Italy; INFN-TIFPA, Trento Institute for Fundamental Physics and Applications, I-38123 Trento, Italy; School of Physics and Astronomy, University of Edinburgh, Peter Guthrie Tait Road, Edinburgh EH9 3FD, UK; MRC Human Genetics Unit, Institute of Genetics and Cancer, University of Edinburgh, Edinburgh EH4 2XU, UK; International Institute for Sustainability with Knotted Chiral Meta Matter (WPI-SKCM^2^), Hiroshima University, Higashi-Hiroshima, Hiroshima 739-8526, Japan

## Abstract

Capturing the physical organisation and dynamics of genomic regions is one of the major open challenges in biology. The kinetoplast DNA (kDNA) is a topologically complex genome, made by thousands of DNA (mini and maxi) circles interlinked into a two-dimensional Olympic network. The organisation and dynamics of these DNA circles are poorly understood. In this paper, we show that dCas9 linked to quantum dots (QD) can efficiently label DNA maxicircles and different classes of DNA minicircles in kDNA. We use this method to study the distribution and dynamics of different classes of DNA minicircles within the network. We discover that maxicircles display a preference to localise at the periphery of the network and that they undergo subdiffusive dynamics. By using simulations, we discover that this peripheral localisation of maxicircles may contribute to the bucking of the structure in solution. Finally, by tracking the dynamics of the QDs we can also quantify the effective network stiffness, confirming previous indirect estimations via AFM. Our method could be used more generally to quantify the location, dynamics, and material properties of genomic regions in other complex genomes, such as that of bacteria, and to study their behaviour in the presence of DNA-binding proteins.

## Graphical Abstract

**Graphical Abstract:**
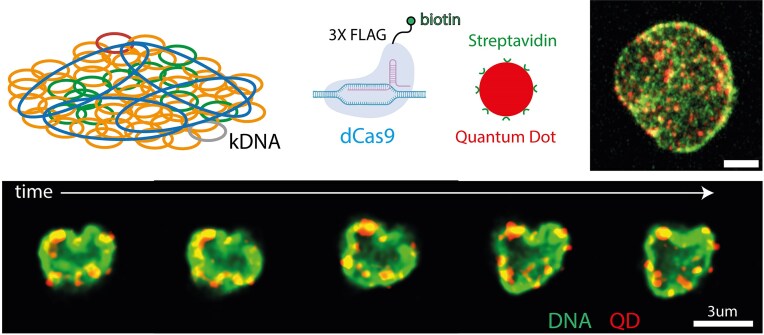

## Introduction

Understanding the spatial organisation of complex and large genomes is currently one of the biggest challenges in biology and biophysics [1, 2]. The kinetoplast DNA (kDNA), the mitochondrial genome of parasites of the class *Kinetoplastida*, such as trypanosomes, is a large (∼10–100 Mbp) complex genome made of interlinked DNA circles. Historically, trypanosomes and the kDNA more specifically, have been at the centre of active research due to its role in pan-genomic RNA editing [3–5]. More recently, the kDNA has also been studied by the polymer physics and topology community as it is the archetype of a so-called ‘Olympic network’, i.e. a structure formed by thousands of topologically concatenated rings [6–15]. Such structures are rare because challenging to controllably synthesise in the lab [16–18].

There are many open questions on the self-assembly, replication and structure of kDNAs [19–26]. For instance, *Crithidia fasciculata* (*C. fasciculata*) kDNA is made of around 5000 short minicircles (2.5 kbp) that are split in 18 genetic classes [14], and of 24 longer maxicircles, around 30 kbp each. While the biological role of these classes of DNA circles in encoding for messenger and editing guide RNAs is known, there are no quantitative measurements of the spatial location of individual genetic classes and whether they are segregated within the network or uniformly distributed [27]. The mechanisms of kDNA replication and reorganisation of maxi and minicircles are intriguing: during replication, minicircles are decatenated, replicated, and reattached to the periphery, while maxicircles do not decatenate from the network and redistribute inside the kDNA with the help of the enzymes [28–30]. The ‘nabelschnur’ structure [30], likely formed by maxicircles, is found to be important for creating a structure connecting the daughter cells during kDNA replication. Thus, kDNA maxicircles may have significant structural role in achieving the elongation and segregation of the kDNA [30]. However, the organisation of maxicircles within the network remains unknown and challenging to quantify.

**Figure 1.**
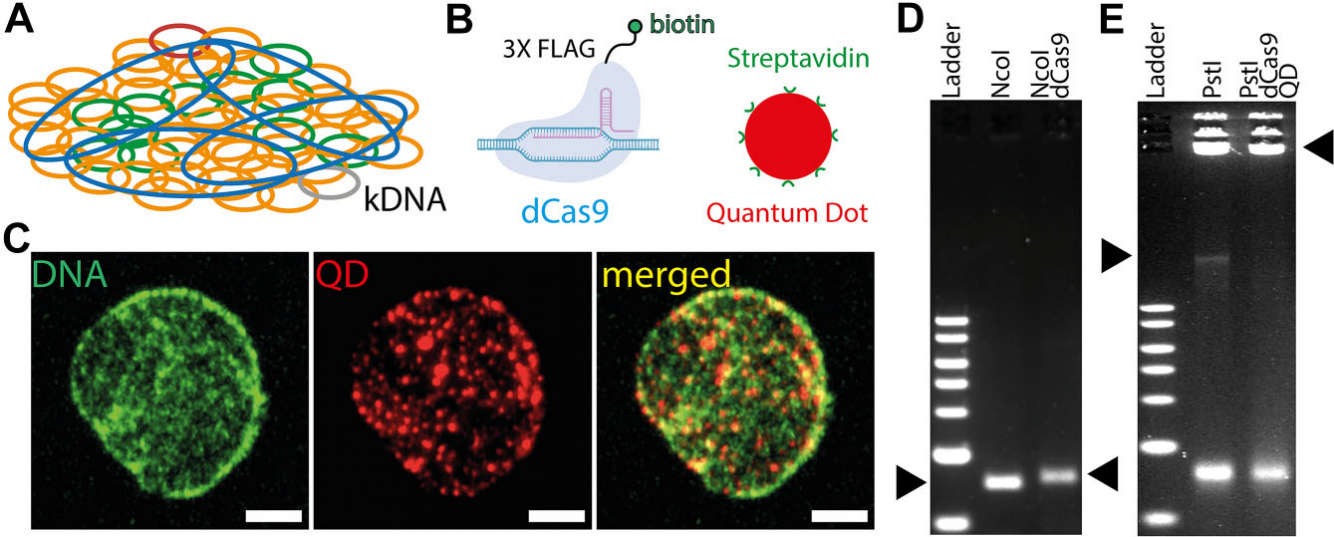
dCas9-labelling of kDNA sequences *in vitro*. (**A**) Sketch of *C. fasciculata* kDNA showing major class minicircles in orange, minor class in green and maxicircles in blue. (**B**) To visualise the different classes of DNA circles we employ dCas9 and streptavidin-coated Quantum Dots (Qdot655). (**C**) Representative image of a kDNA structure with major minicircle class labelled by Qdot655. (**D**) Agarose gel electrophoresis of control and dCas9-bound minicircles, both cleaved with NcoI. The dCas9-bound minicircles shift upward in the gel because heavier than the control. (**E**) Gel of kDNA maxicircles cleaved with PstI (left arrow). When dCas9 proteins are targeting the maxicircles and are bound by Qdot655, the maxicircles form large complexes that remain stuck in the wells with the uncleaved part of the kDNA.

In this paper, we employ catalytically dead Cas9 (dCas9) proteins tagged using quantum dots (QDs) as physical beacons to identify target DNA sequences in *C. fasciculata* kDNA and to quantify their spatial location and dynamics within the network *in vitro* (Fig. 1A–C). We discover that maxicircles are enriched at the periphery, the major minicircle class is mildly depleted at the kDNA rim, while the minor class of minicircles are uniformly dispersed within the network. Through computer simulations, we provide evidence that the location of maxicircles at the periphery and their linking to the minicircles contributes to the buckling of kDNA in solution, as seen in experiments [8, 10], and may be sufficient to cause it. Additionally, we are able to track the dynamics of individual sequences within kDNA maxicircles and their relative displacements. We discover that the dynamics of the maxicircles within the kDNA display a subdiffusive, correlated behaviour that reflects the high level of entanglement. Coarse-grained simulations of the system suggest that the QD dynamics follow that of the minicircle for short timescales. This, joined with the relatively local diffusion of the QD on the kDNA, allows us to use these measurements to estimate the effective stiffness of the kDNA network, which are in line with previous indirect estimations from AFM images [13].

We argue that our method could be used more broadly to quantify the static and dynamic behaviour of specific, individual genomic regions within complex genomes, also in presence of DNA binding proteins such as transcription factors, and ultimately inform their material properties and dynamics.

## Materials and methods

### kDNA and dCas9 validation

Kinetoplast DNA was purchased from Inspiralis (https://www.inspiralis.com) and was purified from *C. fasciculata* using the method in Ref. [31]. CRISPR RNA (crRNA) target sequences in maxicircles and minicircles were identified using the ChopChop online tool (https://chopchop.cbu.uib.no/) using the sequences we obtained in Ref. [14]. ChopChop is a widely used web-based tool for selecting optimal CRISPR/Cas9 target sites: it generates and ranks guide RNA sequences based on conventional criteria such as GC content and self-complementarity, as well as additional factors like predicted efficiency and potential off-target effects. We have always selected three top ranked sequences for our experiments with mini and maxicircles and we specifically used single nucleotide polymorphism and deletion/insertion polymorphism from maxicircles and minicircles to carefully select target sites that avoid sequence overlap and crosstalk. The sgRNA was purchased from Integrated DNA Technologies (IDT) and was assembled with crRNA and universal trans-activating CRISPR RNA (tracrRNA) following the instructions provided by IDT. To assemble the sgRNA structure, 10 μM of crRNA and 10 μM of tracrRNA were mixed in IDT duplex buffer. The mixture was heated to 95^○^C for 5 s and then allowed to cool down on ice for 1 h. The sgRNA samples were aliquoted and stored at –20^○^C. The crRNA targets for kDNA maxicircles (24398 bp) are AGAGGCATCGAAGGATTGAGGGG (seq:5393), TTGAACGAGAATCCTGTATGCGG (seq:23384), AGGTACAACACCATAACACAGGG (seq:13854). The targets for the major minicircle class (2525 base pairs) is GGGCCGAGTGTTCTTGCACGAGG (seq:1678), while for the minor minicircle class (2538 base pairs) is CCGTCGGCAGAAATAGACCTGGG (seq:1727).

To validate the binding of dCas9 to kDNA maxicircles and minicircles, 400 nM of dCas9 was incubated with 800 nM of specific sgRNA (targeting three sites in the maxicircle and one site in both major and minor minicircle classes) in 1X NEB r3.1 buffer at 27^○^C for 30 min. We then mixed the sgRNA–dCas9 complex with 500 ng of kDNA and incubated at 37^○^C for 4 h. The samples were digested with NcoI (for major class minicircles) and with BamHI (for minor class minicircles) at 37^○^C for 60 min (see ‘Results’ section).

For the maxicircles, the binding of dCas9 would not yield a clear shift due to the size of the maxicircles and gel resolution. For this reason, we prepared the binding validation assay as follows: 500 ng of kDNA was mixed with sgRNA-dCas9 complex in 1X NEB r3.1 buffer. The mixture was incubated at 37^○^C for 4 h and then the sample was purified by agarose gel filtration and digested with 1 μL of PstI at 37^○^C for 60 min. Finally, before gel analysis, the maxicircle-digested sample was incubated with 10 nM of Qdot655 Streptavidin Conjugate (Thermo Fisher) at 37^○^C for 5 min, and then analyzed by 1% agarose gel (see Fig. 1D and E and ‘Results’ section).

### Microscopy of dCas9-kDNA-Qdot655 samples

To prepare a dCas9-kDNA–Qdot 655 complex, 400nM dCas9 was first mixed with 800nM of sgRNA (three targets) in 1X NEB r3.1 buffer and incubated at 27^○^C for 30 min. The dCas9-RNA samples were pooled together and mixed with 500 ng of kDNA in 1X NEB r3.1 buffer and incubated at 37^○^C for 4 h. All samples were kept on ice after the respective incubation periods prior to the microscopical analysis. The kDNA-dCas9 sample was loaded onto a 1.5% agarose gel and run at 80V for 20 min and recovered in 1x NEB r3.1 as described in detail in our previous paper Ref. [14]. To prepare the samples for fluorescence microscopy, a clean glass coverslip treated with Poly-lysine was used. A 10 μL of recovered kDNA–dCas9 complex in NEB r3.1 buffer was mixed with 1 μL of 10 nM Qdot655 Streptavidin Conjugate and incubated at RT for 5 min. A 5 μL sample was aliquoted into a separate tube and mixed with 1 μL of 10 nM YOYO-1. The mixture was then placed on a clean glass coverslip and sealed for fluorescence microscopy imaging.

The samples were imaged with a Zeiss LSM980 Airyscan2 laser scanning confocal microscope (Zeiss UK, Cambridge). A 63x/1.4 NA oil immersion objective was used, with 488 nm/633 nm excitation lasers for YOYO-1 and Qdot v655, respectively. Single time-point, 32 slice Z-stacks of the individual kDNA structures with dCas9-Qdot 655 were recorded using the Airyscan detector with a pixel size of 35 x 35 x 160 nm. Images were then Airyscan processed using Zen blue 3.5 software (Zeiss).

To capture the real-time videos for dynamics analysis, 1 μl of YOYO-1-stained Qdot655-kDNA sample was suspended in 4 μL of 70% glycerol, pipetted onto a glass coverslip, and sealed with a sticky spacer. Before acquisition, single suspended kDNA structures were carefully focused on the YOYO-1 channel and selected based on their visible outer ring structure. For each condition at least 25 movies were captured. Movies were recorded in confocal mode exciting with the 405 and 488 nm laser simultaneously exciting and capturing YOYO-1 (detector wavelengths 491–610 nm) and Qdot 655 (detector wavelengths 658–755 nm) at 8 fps, with a pixel size of 70 x 70 nm and at least 500 frames per video. Images were later deconvolved using Hugyens Professional 23.10 software (SVI). The fluorescence microscopy images presented in the manuscript were processed in FIJI (National Institutes of Health) and custom written python codes (see below).

**Figure 2.**
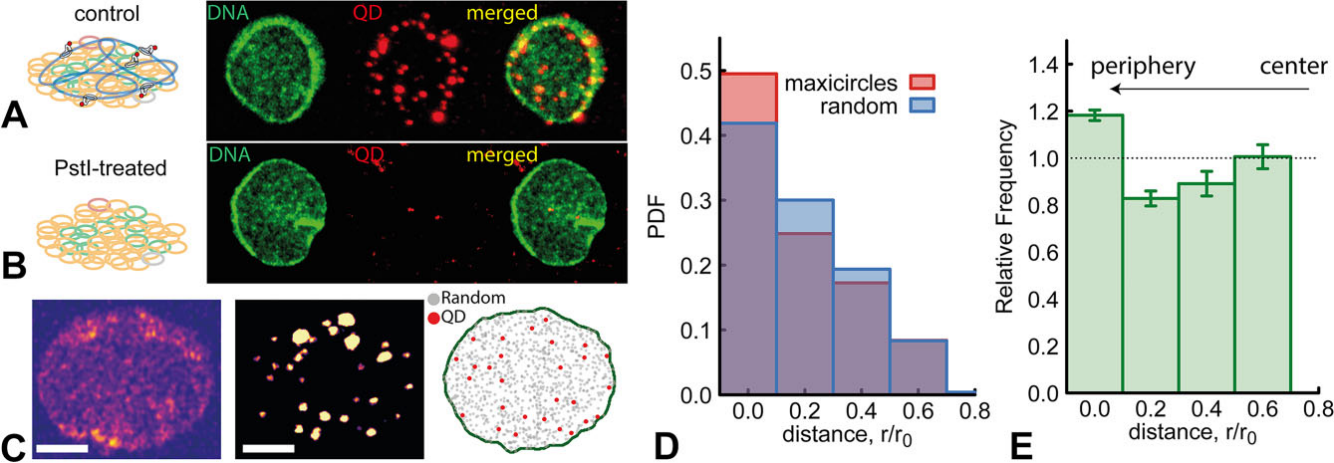
Maxicircles are preferentially located at the periphery. (**A** and **B**) Representative images of YOYO-I labelled kDNA (green) and QDs (red). (**C**) Image analysis pipeline to quantify the distribution of QDs within the kDNA: images are Gaussian blurred and thresholded to detect the boundary of the network and the QDs. The Euclidean 2D distance of each QD from the closest boundary pixel is measured. The same calculation is performed on a simulated kDNA boundary with random points (1000 times the number of Qdots found in the respective kDNA) to obtain a random distribution of distances for individual kDNAs. (**D**) Histograms of distances of QDs (red) and random (blue) points as a function of distance from the kDNA boundary. (**E**) Normalised relative frequency (observed/random) as a function of distance from the kDNA boundary. One can appreciate a significant enrichment of localisations close to the boundary. Error bars represent standard deviation across 13 kDNAs.

Example images obtained with this method are shown in Fig. 2A and B, where the dCas9 is targeting maxicircle sequences in control (Fig. 2A) or PstI-treated (Fig. 2B) kDNA samples. In the latter, since PstI cleaves all maxicircles and the samples are gel purified, we expect and indeed observe no Qdot signal above the noise. Interestingly, we also observed that PstI treatment of the kDNA leads to, on average, smaller kDNAs (see Supplementary Fig. S13), in line with our previous AFM work [14].

### Image analysis

The kDNA images were first processed using Gaussian smoothing to enhance the contrast between the sample and the background. After smoothing, a thresholding was applied to isolate the kDNA. Subsequently, OpenCV’s Canny Edge Detection was employed to extract the boundary pixels of the kDNA. With this method we could robustly exclude any Qdot 655 located outside the kDNA boundary and measure the Euclidean 2D distance of each QD from the nearest kDNA boundary pixel, without the need to assume a simple circular shape.

To quantify the specific location of the Qdots bound to the different kDNA sequences, we compared the distribution of Qdots with a distribution of points (100 times more than the count of Qdots) obtained by randomly sampling the space within the boundary pixels. The observed Qdot655 count per bin was then normalised by this simulated random distribution to reveal any specificity and biases in the location of the sequences (see Fig. 2C).

Tracking of the QD was done from images taken using a spinning-disk confocal microscope. We then used trackpy (github.com/soft-matter/trackpy) on the QD signal to reconstruct their XY movement. To track and the kDNA centre of mass we used an in-house Mathematica code to perform a Gaussian blur and segmentation on the DNA signal, and then to obtain the centroid of the kDNA so to track its XY position during the experiment.

### Molecular dynamics simulations

We performed molecular dynamic (MD) simulations of three different network type. The first system is a network of 604 semi-flexible minicircles catenated with a valence of 3, labelled ‘minicircle only’ (MO). The second has three maxicircles interlinked between themselves and randomly with the minicircle disk, labelled ‘linked diffuse’ (LD). The third has three interlinked maxicircles intertwined with the border of the minicircle disk, labelled ‘linked border’ (LB). In all the configurations, each minicircle is composed of *m*_*mini*_ = 60 beads while the three maxicircles have around *m*_*maxi*_ = 800 beads each. Both circle species have a persistence length of *l*_*p*_ = 4σ and FENE bonds between beads. The minicircle networks are built using NetworkX [32], following the same procedure adopted in ref. [13]. LD and LB networks are obtained by compressing the miniring networks inside a slit and then randomly intertwining the maxicircles with the minicircles network. The initial compression of the MO network is intended to reproduce the *in vivo* conformation of the minirings, a condensed disk-like shape, without relying on further assumptions on its molecular origin. The detailed procedure to construct the networks is reported in the SI (Supplementary Figs S1– S4).

All the systems are evolved using an underdamped Langevin dynamics γ = 0.1 and time step *dt* = 0.01τ_*LJ*_, where τ_*LJ*_ is the characteristic time of the simulation. Equilibration is run for 10^8^ timesteps and production is run for at least 1.5 × 10^9^ timesteps. We analyzed the curvature of the simulated kDNAs by using libIGL for Python and creating a triangulated surface from the COM of the minirings (see ref. [13]).

## Results

### dCas9 can specifically bind to both mini and maxi circles

First, we investigated the binding of dCas9 to mini and maxicircles using Electrophoretic Mobility Shift Analysis (EMSA). We selected three target sites within the kDNA maxicircles, each spaced 5000 base pairs apart, along with a single target in each minicircle class [14]. To assess dCas9 binding to specific DNA types, we first allowed dCas9 to bind to its target sites, followed by digestion of the DNA using sequence-specific enzymes. In both the major and minor minicircle classes, a clear shift in the dCas9-bound DNA bands was observed, confirming sequence-specific, single dCas9 binding (Fig. 1D and E). To distinguish dCas9-bound maxicircles from the control, the complex was further incubated with streptavidin-coated Qdot655, which binds to biotin-labelled dCas9. The disappearance of the maxicircle band in the gel (Fig. 1E) confirmed the specific binding of Quantum Dots to the maxicircles. Interestingly, the presence of dCas9 slightly reduced DNA digestion compared to the protein-free control samples, although this did not affect the outcome of the EMSA assay. This effect is possibly due to the reduced 1D diffusion of restriction enzymes on DNA (see SI, Supplementary Fig. S10). Thus, we confirm that dCas9 can specifically bind mini and maxicircles sequences within the kDNA structure.

### Maxicircles are preferentially located at the periphery of the kDNA

Having confirmed the specific binding of dCas9 to different kDNA circles, we then investigated the spatial distribution of different DNA sequences within the network. To do this, we first targeted three specific maxicircle sequences for dCas9 binding and then incubated the kDNA-dCas9 complex with QDs. Before imaging, we also removed the excess unbound dCas9-QD by gel filtering (as in Ref. [14]). The resulting purified kDNA-dCas9-QD complexes were visualised using confocal fluorescence microscopy. Images were captured separately in the green YOYO-1 channel (labelling the kDNA) and the red 655 channel (labelling the QDs), and later reconstructed into a single composite image to reveal the spatial organisation of maxicircles (Fig. 2A and B). Bright fluorescent QD signal was visible at the periphery of the kDNA, with some signal appearing larger, suggesting potential clustering or co-localisation of dCas9-QD. Additionally, we detected fewer signals located within the central region of the kDNA. To quantify the distribution of QDs, we segmented the DNA signal and reconstructed its boundary. We then segmented and localised the QD signal and placed it within the reconstructed kDNA boundary (Fig. 2C). To compare the distribution of QDs with respect to a uniform distribution, we generated a random deposition process of 100 times more QDs within the same kDNA area. We then computed, for each QD (either randomly placed or real), its distance from the closest boundary pixel, and binned these distances to obtain distributions. The pipeline is represented in Fig. 2C where random localisations are shown in grey and real QD localisations shown in red, all placed within the kDNA boundary (green). The analysis pipeline can be found open access at https://git.ecdf.ed.ac.uk/taplab/kdnart.

In Fig. 2D, we compare the probability density function (PDF) of the random dots (blue boxes) with the one from the real QDs (red boxes). The relative distance, *r*/*r*_0_, represents the binned distance of a localisation from the closest kDNA boundary pixel, normalised by the maximum radial distance of boundary pixels from the centre of the kDNA. In other words, an *r*/*r*_0_ value of 0 indicates that the localisation is at the periphery, while a value of 1 corresponds to a localisation in the middle of the kDNA. The normalisation allows us to sum the data across 13 individual kDNA networks, which have slight variations in their diameter.

To identify any specific enrichment, we then divide (bin by bin) the observed distribution probability (PDF) of the QDs with that of the random, simulated localisation for each kDNA sample. A relative frequency of 1 in a given bin indicates that we statistically find as many QDs as expected for a random (uniform) distribution (see Fig. 2D). From this relative frequency one can appreciate that the distribution of QD is not uniform as a function of distance from the periphery. Interestingly, there is a significant enhancement within the 20% of kDNA area closest to the periphery and lower than random in the kDNA area closest to the centre. This suggests that the maxicircles have a preference for localising to the periphery of the network. Also, no maxicircle sequence is found within the 20% area closest to the kDNA centre.

We note that kDNA has been suggested to display a network-within-network structure where maxicircles are interlinked among themselves and with other minicircles [33] (reported in in *T. equiperdum*), but there is no evidence of the maxicircles being enriched at the periphery of the kDNA in any organism. Thus, our data represent the first of such evidence in *C. fasciculata*.

We also stress that while maxicircles appear enriched at the periphery, major and minor minicircle classes that we tested do not display a significant enrichment, suggesting a more homogeneous distribution.

### A peripheral distribution of linked maxicircles is sufficient to cause kDNA buckling.

In solution, kDNA assumes a buckled shape with positive absolute mean curvature that resembles a showercap [8, 10, 11]. This is surprising because a thermal sheet with no pre-stored stress should display a flat shape with overall mean curvature close to zero [34]. It has been hypothesised that either the chirality of the linkages [35] or redundant linking at the periphery [13] may be the reason behind the overall shape of the network.

In our previous work [14], we found qualitative evidence that some maxicircles were located at the periphery of the network. In the previous section, we have quantitatively demonstrated that indeed maxicircles display a preference to be located at the periphery kDNAs. In this section, we now ask if the peripheral positioning of the maxicircles may affect the overall curvature of the network.

To do this we performed MDs simulations in LAMMPS [36] of model kDNA networks with and without maxicircles (see ‘Materials and methods’ and SI). These networks are created using planar graphs and the DNA circles are modelled as coarse-grained ring polymers made by beads connected by FENE springs [37]. The minicircles are interlinked to each other with random chirality and valence 3 [38]. The maxicircles are randomly intertwined within a planar minicircles network compressed to resemble the disk-shape assumed by kDNA *in vivo*. This is done under two different hypotheses: (i) Maxicircles are found at the border (LB networks) or (ii) Maxicircles are found within the kDNA disk (LD networks). In both cases, the networks are constructed by first confining the position of maxicircles within the kDNA through external potentials (later removed), and then slowly introducing a steric interaction between minicircles and maxicircles. This, together with FENE bonds in between bonded beads, ensures that the topology of the kDNA network is conserved [37]. After initialisation, all constraints are removed and the system is equilibrated to relax any stress introduced during the preparation. We then perform production runs for at least 1.5 × 10^9^ timesteps. To verify that the system is truly in equilibrium, we checked that 95% of the values of the kDNA radius of gyration are within two standard deviations from the mean, i.e. that the size of the kDNA is not evolving or drifting during the production run.

**Figure 3.**
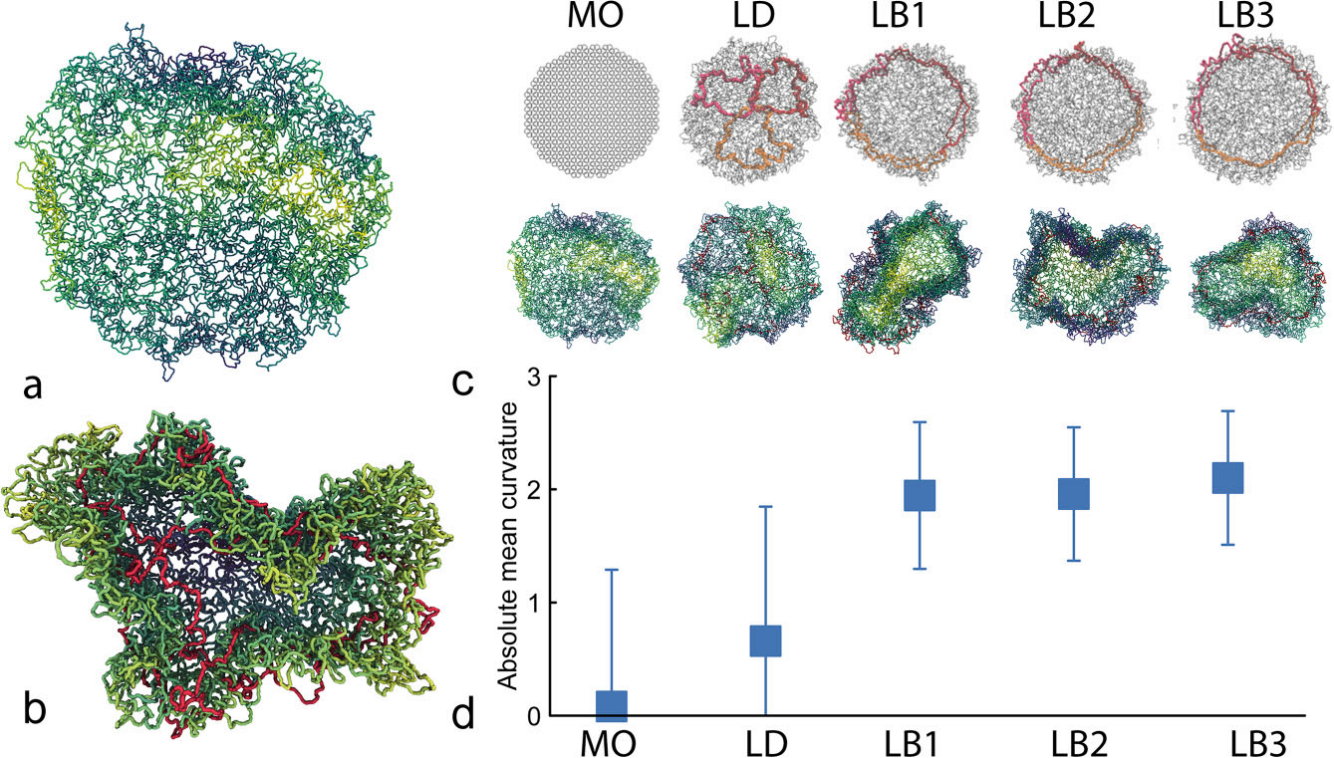
MD simulations of kDNA suggest peripheral maxicircles induce buckling. (**A** and **B**) Representative snapshots of equilibrium configurations of kDNA networks without (a) and with (b) maxicircles. (**C**) Snapshots of initial and equilibrium conformations of different kDNA topologies. (MO = minicircle only, LD = linked diffuse, LB1,2,3 = linked border, three different replicas). (**D**) Absolute mean curvature for the different kDNA topologies. The LB models display the largest mean curvature, reflective of buckling induced by the maxicircles linked at the border.

From our simulations, we computed the mean curvature of the networks (see Methods and SI, Supplementary Figs S5 and S6) and obtained the results in Fig. 3B. Configurations consisting of only minicircles display zero mean curvature compatible with a saddle shape. This is consistent with previous simulations of flat membranes [34] and kDNA models [13, 39]. Adding maxicircles at random locations inside the disc causes an increment of the absolute average mean curvature, but the system can still frequently jump between positive and negative mean curvatures (see SI, Supplementary Figs S6 and S7). Interestingly, only when we placed the maxicircles along the border we observed net positive mean curvatures compatible with the experimentally observed buckled shapes [8]. We argue that this is because they constrain the total perimeter of the disc, acting as an elastic band running along the border of the kDNA [13]. Arguably, trypanosome species that have a different network replication and organisation, like *T. brucei*, may retain the a saddle-like shape in solution. We hope to test this hypothesis in the near future.

### Dynamics of maxicircles sequences within the kDNA

**Figure 4.**
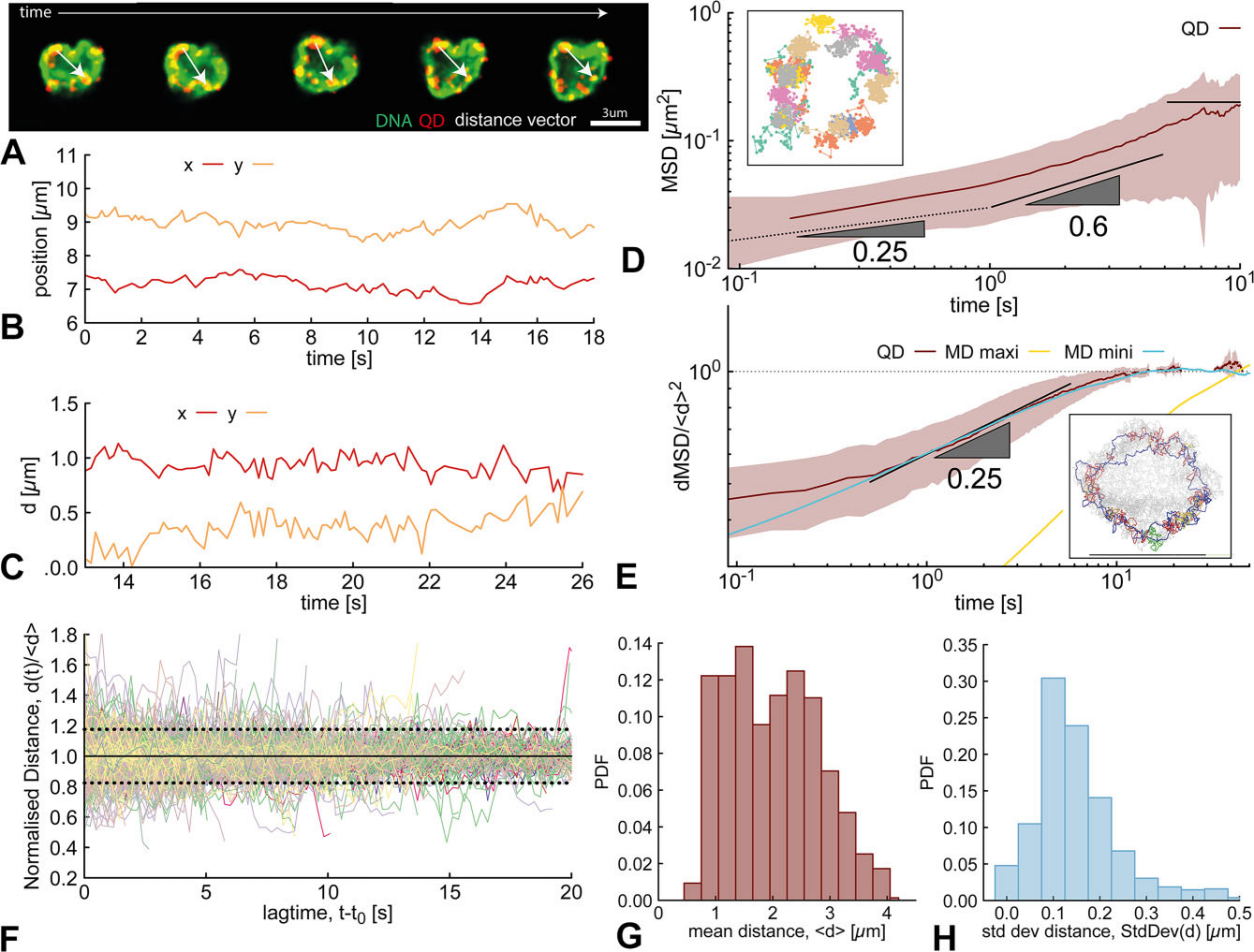
Dynamics of maxicircle sequences. (**A**) Five snapshots taken with a spinning-disk confocal microscope of a fluctuating kDNA in solution at sequential times. DNA signal in green, QD signal in red. The white arrow indicates the distance vector between two QDs. (**B**) Example of the position of one QD (x and y coordinates) over time. (**C**) Example of the distance vector between two QD (x and y components) over time. (**D**) Mean squared displacement (MSD) of the QDs in the frame of reference of the kDNA COM (*g*_2_(*t*)). (**E**) Mean squared displacement of the distance vector ${\rm\boldsymbol$d$}$ between pairs of QDs and normalised by the mean distance (squared) of each pair, dMSD/〈*d*〉^2^, averaged across pairs and initial times. Blue and yellow lines are from simulations tracking maxicircles and minicircles, respectively. In the inset we show maxicircles in blue and minicircles threaded by the maxicircles in colours. (**F**) Normalised distance between pairs of QDs. Dashed lines represent one standard deviation from the mean. (**G**) Distribution of mean distances between QDs. (**H**) Distribution of standard deviations of distances between QDs.

The main advantage of labelling DNA circles sequences with dCas9-QDs is that we can quantify their dynamics in real time. We employed a fast spinning-disk confocal to record two-colour movies at >10 fps of labelled kDNAs and QDs diffusing in a glycerol solution, optimised to record the dynamics of the kDNA (see ‘Materials and methods’, and Fig. 4A).

The QD signal was processed with a tracking algorithm (see Methods) which allowed us to obtain the 2D location of each QD over time (see fig. 4B). To remove the global translation of the kDNA, we also tracked the position of the kDNA centre of mass (COM) using the DNA signal. We then computed the mean squared displacement of the single QDs in the frame of reference of the kDNA COM, denoted here as $\boldsymbol{r}_{CM}$, i.e.


\begin{eqnarray*}
g_2(t) = \langle [(\boldsymbol{r}_i(t+t_0) - \boldsymbol{r}_{CM}(t+t_0)) - (\boldsymbol{r}_i(t_0) - \boldsymbol{r}_{CM}(t_0)) ]^2 \rangle \, .
\end{eqnarray*}

As one can appreciate from Fig. 4D, *g*2(*t*) displays two subdiffusive regimes, until it reaches a long time plateau at *t* > 10 s. The subdiffusive, and hence correlated, motion of QDs bound to the maxicircles is to be expected, as the topological links create a correlation similar to that of bonded segments in tethered membranes [34]. However, in classic Rouse dynamics of single polymers, one expects *g*2 to plateau around the size of the polymer $R_g^2$. In this case, instead, we observe a slower-than-Rouse dynamics [40] (*g*_2_ ∼ *t*^α^, α < 0.5) and a transition to the plateau when QDs have, on average, diffused less than 0.5 μm, a distance far smaller than the size of the kDNA (around 4 μm). Comparison with simulations suggest that this behaviour could be ascribed to QDs following a dynamics in which they remain entangled with minicircles for some time, before threading through minicircles. This entails that *g*_2_ of the QDs would follow the dynamics of minicircles at short times, and then progressively switch to that of maxicircles at longer times. Eventually, we expect *g*_2_ to reach a plateau describing the maximum (squared) distance of maxicircles from the kDNA COM. To check this scenario, we computed *g*_2_ for the linked-border simulations and by aligning the trajectory (removing translations and rotations) for both maxicircles and a set of minicircles threaded by maxicircles. The results, reported in Fig. 4 and the SI (Supplementary Figs S8 and S9), show that α_*sim*_ ∼ 0.25 for minicircles, and ∼ 0.5 for maxicircles, supporting the argument that, while bound to maxicircles, QDs may be marginally entangled with minicircles.

Additionally, we used our time-resolved imaging to infer the elastic properties of the network in a manner akin to that done for polymeric networks [41]. We can interpret *g*_2_(*t*) as reflecting the fluctuations of DNA maxicircles sequences within a tethered, or crosslinked, structure. According to the equipartition theorem, at large times we expect *g*_2_(*t*) to be proportional to the thermal energy and inversely proportional to the effective kDNA stiffness i.e. *g*_2_(*t* → ∞) = 2*k*_*B*_*T*/κ. In Fig. 4D, one can appreciate that the extent of these fluctuations are limited at around 0.2 μm^2^, and we thus obtain:


\begin{eqnarray*}
\kappa = \dfrac{2 k_B T}{0.2 \mu m^2} = 0.04 \dfrac{pN}{\mu m} \, ,
\end{eqnarray*}

which is in line (although slightly smaller than) with our previous estimations based on AFM images of 0.1 pN/μm [13, 14]. We hypothesise that by removing (or linearising) some of the mini-circle classes forming the kDNA, we should expect a smaller effective stiffness.

Another way to analyze the dynamics of the QDs that has the benefit of naturally removing the roto-translational motion of the kDNA is to consider the distance vector between pairs of QDs as $\boldsymbol{d}_{ij} (t)= \boldsymbol{r}_i(t) - \boldsymbol{r}_j(t)$ (see Fig. 4A and C). We then compute its MSD as


\begin{eqnarray*}
\textrm {dMSD}(t) = \langle \left[ \boldsymbol{d}_{ij}(t-t_0) - \boldsymbol{d}_{ij}(t_0)\right]^2 \rangle \, ,
\end{eqnarray*}

where the average is performed over initial times *t*_0_ and over pairs of QDs. The MSD of the distance vector, dMSD, is a quantity typically measured when tracking genomic sites *in vivo* [42, 43] and could therefore be compared to experimental values of genome dynamics within the cell. As one can appreciate in Fig. 4E, dMSD displays a strong subdiffusive regime, where dMSD(t) ∼ *t*^0.25^, somewhat slower than the exponent expected for tethered membranes [34].

The dynamics of the distance between QDs can also yield information on the network stiffness. Indeed, by plotting all the QDs distance traces as a function of lag-time, i.e. $d(t)=|{\rm\boldsymbol$d$}(t-t_0)|$, and normalised by the mean distance 〈*d*〉 we observe that they are all contained within 1 ± 0.2 (Fig. 4F). More precisely, we obtain an average mean distance between QDs of $\bar{\langle d \rangle } = 2.16$ μm and an average standard deviation of σ_〈*d*〉_ = 0.17 μm (see Fig. 4G and H). Again from the equipartition theorem we expect that the dynamics of the QDs’ distance should be equivalent to that of points connected by an effective spring with stiffness $2 k_B T/ \sigma _{\langle d \rangle }^2 = 0.28$ pN/μm, which also in excellent agreement with our previous estimate [13]. We argue that measuring the dynamics and the effective stiffness experienced by different DNA sequences, we will be able to infer inhomogeneous structures within the kDNA network. In turn, we could potentially extend this analysis to other complex genomes, therefore mapping their elastic properties *in situ*.

In summary, this is the first time we could obtain direct measurements on the stiffness of kDNA networks using time-resolved imaging and have found that these measurements are in broad agreement with the estimations from AFM images (which were only based on the network structure). We argue that our dynamic data could be directly compared with simulations of tethered and topologically interlocked membranes [34] and to previous microscopy kDNA experiments [8, 10] (see Supplementary Fig. S12) to better understand the dynamic scaling of Olympic-like networks compared with traditional crosslinked ones.

## Discussion

Understanding the spatial organisation of complex genomes is a question that is fascinating and ubiquitous. We have here shown that QD-labelled dCas9 can be used as ‘beacons’ to map the location and dynamics of DNA sequences in a topologically complex genome such as the kDNA (Fig. 1).

We used this method to map the location of kDNA maxicircles, which have previously been hypothesised to populate the outer part of kDNA networks [14, 30]. Indeed, using our method we have quantitatively demonstrated that maxicircles are preferentially located at the periphery (Fig. 2). We note that while the network-within-network interlinked structure of maxi and mini circles has been observed before in *T. equiperdum* [33], the localisation of maxicircles at the periphery in *C. fasciculata* had not been appreciated so far. Importantly, we also showed (see SI, Supplementary Figs S10 and S11) that major and minor minicircle classes do not display a strong enrichment at the periphery like maxicircles do, and instead appear more uniformly distributed over the kDNA network. Though there is no solid experimental evidence for how the mini- and maxi-circles are redistributed within the kDNA during replication, we argue that the positioning of maxicircles at the periphery may play a structural function, for instance in establishing correct nabelschnur structure [30] and in the partitioning of the kDNA to the daughter cells.

We then asked if the peripheral location (and interlinking [14]) of maxicircles played a role in determining the buckled shape of kDNA in solution [8]. To answer this question we performed MD simulations of different kDNA topologies, including without maxicircles, or with maxicircles linked throughout the kDNA or only at the periphery. We observed that in the latter case the network buckles and displays the largest mean curvature (Fig. 3). We therefore argue that the observed buckling of kDNA in solution is not due to the chiral arrangement of the minicircle links, but it is instead due to the positioning of the maxicircles.

Finally, we used our dCas9 labelling technique to track the dynamics of maxicircle sequences (Fig. 4). We discovered a largely subdiffusive dynamics, slower than the dynamics seen in simulations of tethered membranes [34]. By measuring the fluctuations of the dCas9 proteins with respect to either the centre-of-mass (COM) of the kDNA or with respect to each other, we obtained direct measurements of the kDNA network effective stiffness finding values κ ≃ 0.06–0.4 pN/μm, which are in excellent agreement with our previous estimate of 0.1 pN/μm based on AFM images alone [13, 14]. This measurement confirms a previous hypothesis that the kDNA is an ‘ultra-soft’ 2D polymeric membrane, especially when compared with lipid bilayers or other 2D structures which typically display stiffnesses ≃ 1μ*N*/μ*m*, i.e. 10^6^ times larger.

We expect that our method could be applied to mapping the location and dynamics of DNA sequences in other complex genomes, for instance in ‘genome-in-a-box’ set ups [44] or even DNA origami, and could in turn provide information on the material properties of these structures.

## Supplementary Material

gkaf925_Supplemental_File

## Data Availability

Codes are available on Zenodo at https://doi.org/10.5281/zenodo.16965165.
