# Supplementary material for: Organisation and dynamics of individual DNA segments in topologically complex genomes
Source: Nucleic Acids Res. 2025 Nov 11;53(20):gkaf925. doi: 10.1093/nar/gkaf925 (PMC12604670; doi:10.1093/nar/gkaf925)
Supplement: gkaf925_Supplemental_File [file gkaf925_supplemental_file.pdf]

Organisation and dynamics of individual DNA segments in topologically complex genomes: Supplementary Information

Saminathan Ramakrishnan,[1] Auro Varat Patnaik,[1] Guglielmo Grillo,[2] Luca Tubiana,[2,3] and Davide Michieletto[1,4]

[1]*School of Physics and Astronomy, University of Edinburgh*
[2]*Physics Department, University of Trento, via Sommarive, 14 I-38123 Trento, Italy*
[3]*INFN-TIFPA, Trento Institute for Fundamental Physics and Applications, I-38123 Trento, Italy*
[4]*MRC Human Genetics Unit, Institute of Genetics and Cancer, University of Edinburgh**

MOLECULAR DYNAMICS SIMULATIONS: ADDITIONAL DETAILS

Reduced Kinetoplast Network

A Kinetoplast DNA (kDNA) from trypanosomatid *Crithidia fasciculata* has around 5000 minicircles with $\sim 2.5kbp$ each and around 30 maxicircles with $\sim$ 30 kbp each [1]. Performing molecular simulations at atomistic resolution of this structure is not feasible, and even a standard bead-and-springs model with $\sim$ 7.5 bp per bead would prove very difficult to equilibrate. Since we can assume that the physical properties of the kDNA stem from its topology, and thus that the most important interaction is only the steric one, we map a kDNA to a system of 604 minirings, each made of 60 beads, and 3 maxirings made of approximately 800 beads each. This corresponds to a reduction in scale by a factor of 8 for both the surface and the number of maxi circles. Each bead corresponds to $\sim$ 42.7 bp.

Hamiltonian

Minicircles and maxicircles are modeled through a standard Kremer-Grest model. All beads have diameter σ. Steric interaction between pairs of beads i and j are accounted for by using the Weeks-Chandler-Andersen (WCA) potential:

$$U_{WCA}(i,j) = \begin{cases} 4\epsilon \left[\left(\frac{\sigma}{r_{ij}}\right)^{12} - \left(\frac{\sigma}{r_{ij}}\right)^{6} \right] + \epsilon & \text{if } r_{ij} \le 2^{1/6}\sigma \\ 0 & \text{if } r_{ij} > 2^{1/6}\sigma \end{cases} \quad (1)$$

where $r_{ij} = \|\mathbf{r}_i - \mathbf{r}_j\|$ and $\epsilon = 1$ sets the simulation energy scale

Bonds between pairs of consecutive beads $i, i+1$ are modeled through a finitely extensive non-linear elastic (`FENE`) potential

$$U_{FENE}(i, i{+}1) = \begin{cases} -\frac{kR_0^2}{2} \ln \left[1 - \left(\frac{r_{i,i+1}}{R_0}\right)^2 \right] & \text{if } r_{i,i+1} \le R_0 \\ 0 & \text{if } r_{i,i+1} > R_0 . \end{cases} \quad (2)$$

where the maximum extension of the bond is set to $R_0 = 1.5\sigma$ and its strength to $k = 30.0\epsilon/\sigma^2$.

The bending potential, $U_{bend}(i, i+1, i+2)$ is given by a Kratky-Porod term:

$$U_{bend}(i, i+1, i+2) = \kappa_\theta \left(1 - \frac{\mathbf{t}_i \cdot \mathbf{t}_{i+1}}{||\mathbf{t}_i||||\mathbf{t}_{i+1}||} \right), \quad (3)$$

where $\mathbf{t}_i = \mathbf{r}_{i+1} - \mathbf{r}_i$ is the $i-th$ bond vector, $\kappa_\theta = \frac{k_B T l_p}{\sigma}$ and l_p is the persistence length. We choose $l_p = 4\sigma$, roughly matching the known bending persistence length of dsDNA. In the FENE and Kratky-Porod potentials chain circularization are enforced by setting $n + k = k$, with n the number of beads of a ring.

All simulations are run using an underdamped Langevin dynamics, starting from previously built networks of minicircles and maxicircles.

Network construction

We construct and simulate three different network types. The first, labelled MO (minirings only), is composed of only minirings, and is build following the procedure introduced in [2]. In short, using NetworkX version 3.3 [3], a Python package for the creation, manipulation, and analysis of networks, we build a planar hexagonal lattice patch whose nodes correspond to the center of mass of the minirings. Minirings are then added to the nodes so that each one interlinks with its neighbours through a Hopf-link that is randomly picked to have +1 or −1 sign. This is done by alternating perfectly planar rings with randomly picked distorted rings. The other two network types include three interlocked maxirings that are intertwined with the minirings networks. The maxirings are either placed along the border of the miniring network (LB - Linked Border topology), or randomly within the minirings disk (LD - Linked-Diffusive topology). Both networks are produced by intertwining maxicircles with a flat minicircle network equilibrated inside a slit and subject to a slight radial confinement. This mimics the fact that in vivo the the kDNA is confined and is known to be a flat disk [4]. The procedure is as follows.

Minicircles and maxicircles are first simulated for $10^4\tau_{LJ}$ timesteps of underdamped Langevin dynamics

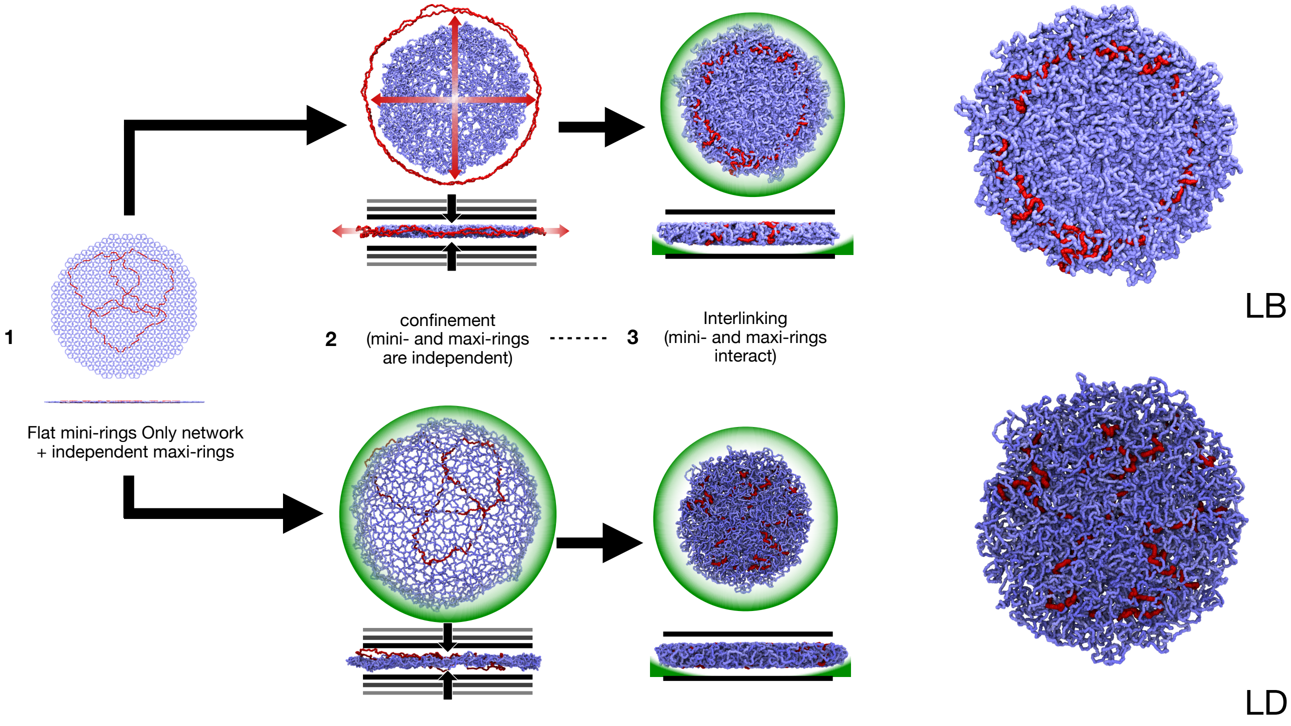

FIG. S1. Top row: assembly steps for “Linked-Border” networks. Bottom row: assembly steps for ”Linked-Diffusive” networks. In step 1 the mini-ring network and the maxi-ring are equilibrated under the same vertical confinement conditions, but without interacting with each other, aside from a spring that keeps the maxi-ring system within the minirings. Black horizontal lines represent walls on z. Green shaded area is a pictorial depiction of the harmonic potential constraining the maxi-ring system to remain in the same region as the mini-ring network.

while being constrained on the XY plane by two walls along z. The walls are positioned at $\pm 30\sigma$ at $t = 0$ and their distance is progressively reduced until their positions reach $\pm 11\sigma$ at $t = 10^4 \tau_{LJ}$. The walls and the the beads interact through a standard WCA potential. During this time, maxicircles in LD and LB networks are subjected to different ad-hoc potentials to position them inside the minicircles network or near its border respectively, see Fig. S1, step 2. In the LD case, they are subjected to a potential $V_{LD}(\mathbf{r}) = -\exp^{-(\sqrt{2}R_g^{mini}(t) - \|\mathbf{r}\|)}\,\hat{\mathbf{r}}$. In the LB case, the maxicircles are instead stretched by a potential $V_{LB}(\mathbf{r}) = \exp^{-(\sigma + 0.0139\sigma \cdot t - \|\mathbf{r}\|)}\,\hat{\mathbf{r}}$. During this equilibration step mini-rings and maxi-rings systems are invisible to each other aside for the confining potential based on the minicircles' radius of gyration.

Following this step we add a harmonic constraint on the radius of gyration of both the minicircles and maxicircles in order to further shrink the network. This constraint is:

$$V_{HC}(t) = K_{HC}\left(r_g(t) - r_{target}^{sys}\right)^2 \tag{4}$$

where $K_{HC} = 20\epsilon/\sigma^2$ and r_{target}^{sys} depends on the specific system (maxicircles vs minicircles) and network. Specifically, we choose $r_{target}^{mini} = r_{target}^{maxi} = 50\sigma$ for both maxi- and mini-circles in LD, $r_{target}^{mini} = 70\sigma$, $r_{target}^{maxi} = 90\sigma$ for LB1, $r_{target}^{mini} = 50\sigma$, $r_{target}^{maxi} = 60\sigma$ for LB2 and LB3. Each system is equilibrated for $5 \cdot 10^3 \tau_{LJ}$ under these conditions (which include the previous constraint to confine the maxicircle to lie within the minicircles network) (step 3 in Fig. S1).

Finally, we let minicircles and maxicircles interact directly by adding a soft potential whose strength increases linearly. This introduces an excluded volume effect between minicircles and maxicircles and thus topologically intertwines the maxicircles within the minicircle network. The soft potential is

$$U(i,j,t) = \begin{cases} A(t)\left[1 + \cos\left(\frac{\pi r_{ij}}{r_c}\right)\right] & \text{if} \quad r_{ij} > r_c, \\ 0 & \text{otherwise,} \end{cases} \tag{5}$$

$r_c = 1.0$ and $A(t)$ rises linearly from 0 to 100 in $0.5 \times 10^4 \tau_{LJ}$ timesteps. During this step minicircles are kept fixed and only the maxicircles are evolved. The final topology is obtained by replacing the soft interaction with a Weeks-Chandler-Andersen potential between all beads.

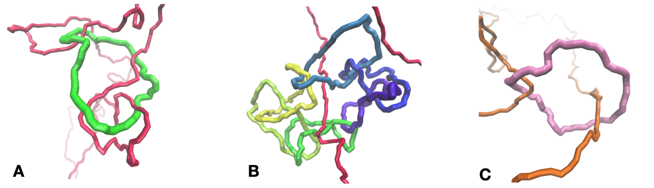

FIG. S2. (**A**) Maxicircle linked with a minicircle. (**B**) Maxicircle linked to a mini-6-catenane without being linked to the minicircles. (**C**) Maxicircles network linked with a minicircle.

Production runs

All the systems are evolved using an underdamped Langevin dynamics with time step $dt = 0.01\tau_{LJ}$, where τ_{LJ} is the characteristic time of the simulation, and diffusion coefficient $\gamma = 0.1$. At the end of the building procedure, all networks are equilibrated for $1 \cdot 10^6 \tau_{LJ}$ in bulk. Production simulations are then run for at least $1.2 \times 10^7 \tau_{LJ}$.

Hopf-links vs higher-order links in simulated networks

During the generation of the topology (*Section "Network construction"*), the maxicircles are embedded randomly with the rest of the network by slowly adding the excluded volume interaction between them and the minicircles while the whole system is confined. The resulting linking between maxi- and minicircles is not predetermined and emerges due to the excluded volume interactions and the confinement.
We design a procedure to analyse the most common form of topological linking between mini- and maxicircles, which exploits the underlying regular hexagonal lattice structure of the minicircles and the circular 3-catenane configuration of the maxicircles.
The most common links are of the following type:

1. Normal "first order" links between a single minicircle and a maxicircle (Gauss linking number != 0, *Figure* S2, A);

2. Second-order links between a maxicircle and a circular "mini-6-catenane" formed by 6 minicircles (*Figure* S2, B);

3. Second-order links between the "maxi-3-catenane" formed by the maxicircles and either a minicircle or a mini-6-catenane (*Figure* S2, C).

Links of type 1 can be obtained by computing the Gaussian linking number[5] between two circles γ_1 and γ_2:

$$L_k(\gamma_1, \gamma_2) = \frac{1}{4\pi} \oint_{\gamma_1} \oint_{\gamma_2} \frac{\boldsymbol{r}_1 - \boldsymbol{r}_2}{|\boldsymbol{r}_1 - \boldsymbol{r}_2|^3} \cdot (d\boldsymbol{r}_1 \times d\boldsymbol{r}_2).$$

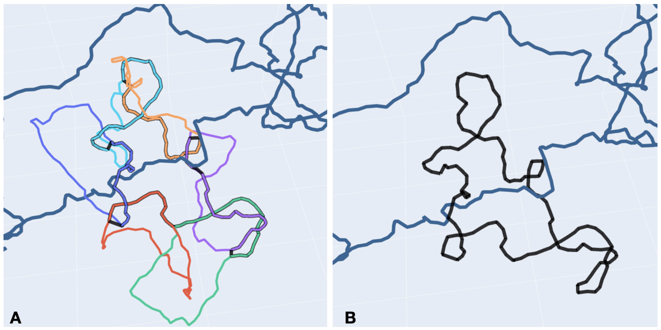

FIG. S3. (**A**) Maxicircle linked with a mini-6-catenane. Only the six minicircles forming the mini-6-catenane are shown. In black, the path we constructed by connecting the edges of the minicircles and the segment of minimal distance between them. (**B**) Maxicircle and the circle corresponding to the mini-6-catenante.

To identify links of type 2, we construct a closed path that traverses all rings in a circular catenane, ensuring no mini-6-catenane minicircle is linked to any maxicircle. First, we apply kymoknot's rectification algorithm[6] to straighten each ring without altering its topology. Next, for every linked pair of rings, we find the closest points and connect them with an edge. Starting from an arbitrary bead on any ring, we follow the contour of the ring until we reach one of these connecting edges. We then jump to the adjacent ring and repeat the process. Once we obtain a closed path, the cycle is complete. Since ring orientations are fixed by bead ordering, the path is unambiguous. We then use the gaussian linking number to detect when a maxicircle is linked with the equivalent circle but not with any of the minicircles constituting the path. An example is shown in Figure S3, where the black path connects the minicircles of a mini-6-catenane.

We employ an equivalent procedure to identify type 3 links: a closed path is constructed from the maxi-3-catenate and the Gaussian linking number is used to identify those minicircles that are linked with the maxi-3-catenate but not to a single maxicircle.
In *Figure* S4, we show the number of links for each type in the four cases considered. Links detectable by Gaussian linking are the majority of the links detected, while "mini-6-catenane" and "maxi-3-catenane" are rare.

Computation of mean and gaussian curvature on meshes

The simulated networks were mapped on triangular meshes using the same scheme adopted in [2], leveraging the fact that the minicircles networks are hexagonal. The meshes are analized using the libIGL (v. 2.2.1) Python package [7]. We compute the total mean curvature as the

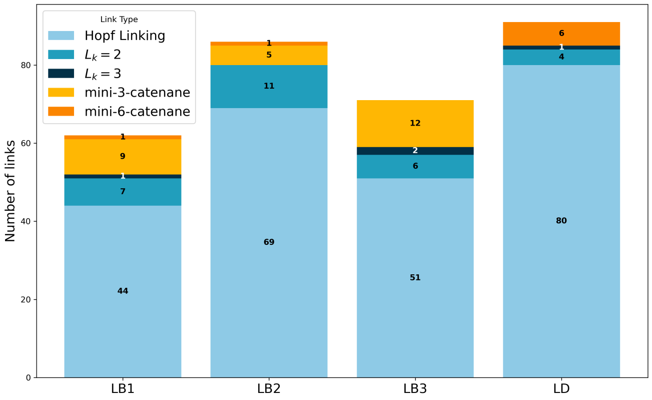

FIG. S4. Number of link types for the four cases considered. Links detectable by Gaussian linking represent the majority of the links.

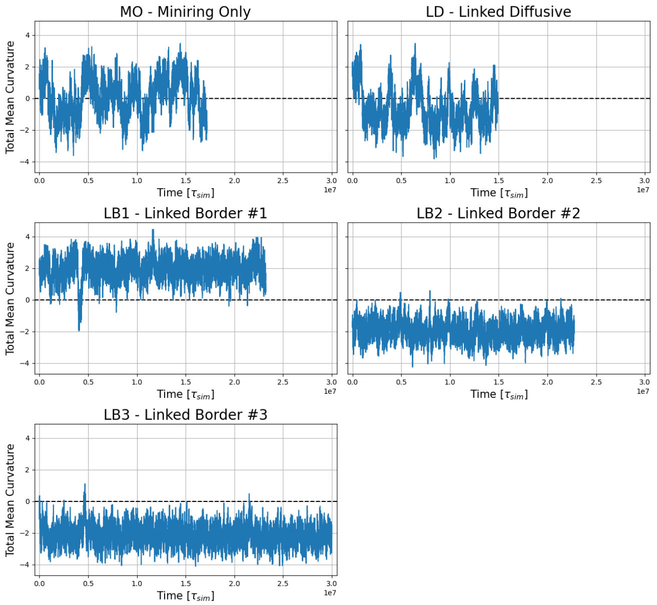

FIG. S5. Time evolution of the total mean curvature for all the configuration examined. The presence of maxicricles on the order (LB1, LB2, LB3), induces a net curvature that is not observed in the other cases (MO, LD).

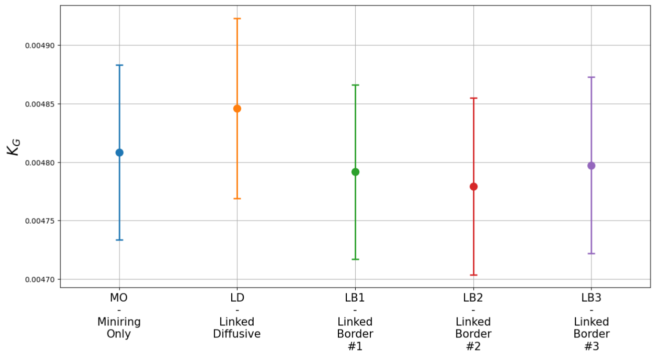

FIG. S6. Average integral Gaussian curvature for the configuration examined. No significant difference is found between the different configurations.

sum of the mean curvature of all the facets, defined as

$$\Sigma = \sum_{i=1}^{N} \frac{1}{2}(K_{1,i} + K_{2,i}) \tag{6}$$

where N is the number of facets, and $K_{1,i}$ $K_{2,i}$ are the principal curvature axes on facet i. To obtain K_1 and K_2 we use the function `principal_curvature` from libIGL.

The Discrete Gaussian curvature can be computed as well using function `gaussian_curvature`, which returns the discrete approximation of the gaussian curvature on each vertex:

$$K_G(i)\,\mathrm{dA}_i = 2\pi - \sum_{k=1}^{N_f} \theta_k(i), \tag{7}$$

where N_f is the number of facets incident on vertex i and $\theta_k(i)$ is the angle in vertex i of the k-th triangle, and dA_i is the voronoi area around vertex i. The integral of $K_G(i)$ over the whole surface is given by:

$$K_G = \frac{\sum_i K_G(i)\mathrm{dA}_i}{\sum_i \mathrm{dA}_i}. \tag{8}$$

We notice that since the minicircles mesh is fixed across different simulations, irrespective of the distribution of the maxicircles, the results provided by eq. 8 will only depend on the area of the surfaces.

Figure S5 shows the total mean curvature as a function of time. We can observe how the presence of the maxicircles on the border induces a net curvature in a direction.

Figure S6 shows the integral of discrete gaussian curvature K_G over the whole mesh while *Figure* S7 shows its time evolution.

MSD and two point MSD

We approximate the presence of Qdot655 and its interaction with the kDNA in two different ways:

1. by selecting all the beads (MSD) or 16 equally spaced beads (dMSD) on each maxicircle or,

2. by selecting all 60 beads (MSD) or 1 bead (dMSD) on the minicircles threaded by maxicircles (the latter case consists of between 50 and 80 points per simulation).

The second case is used to follow the dynamics of the minicircles at the border and reflects the possibility that the QDs will frequently stop to thread the minicircles.

Using these two different set of points we compute the MSD and two-point dMSD indicated in the paper after having removed rototranslations (see *Section "Alignment"*). For the dMSD, pair of points are selected in

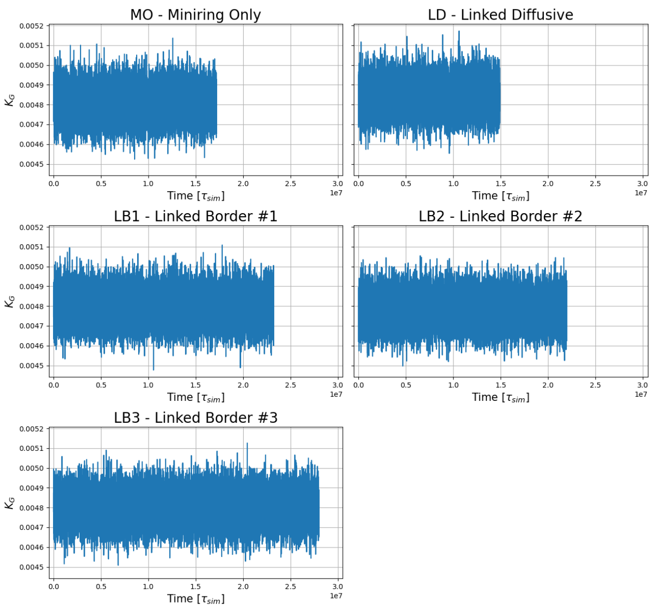

FIG. S7. Time evolution of integral Gaussian curvature for all the configurations examined.

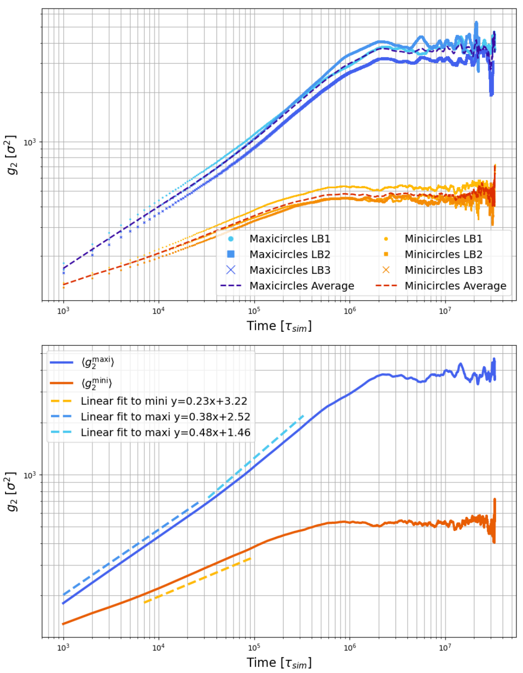

FIG. S8. (ABOVE) Mean Squared Displacement (MSD) for all the configurations with maxicircles located on the border. The dashed lines represent the average curves. (BELOW) MSD of the average curves for both minicircles and maxicircles with the corresponding fits.

such a way that they correspond to different maxicircles and their curve normalised by the expected average value for completely decorrelated times:

$$\lim_{\tau \to \infty} \langle (X(t+\tau) - X(t))^2 \rangle = 2\langle X^2 \rangle - 2\langle X \rangle^2 \qquad (9)$$

Figure S8 shows the MSD computed for all the configuration examined, including both maxicircles and minicircles threaded by the maxicircles. The average MSD scales as $\sim t^{0.23}$ for minicircles and as $\sim t^{0.38}$ and $t^{0.48}$ maxicircles.

Similarly, *Figure* S9 shows the normalized dMSD computed for the same set of configurations. The average dMSD scales as $\sim t^{0.22}$ for minicircles and as $\sim t^{0.43}$ for maxicircles.

Alignment

Since the experimental observations of the kDNA show little rotations, and only on the XY plane, while the simulations are long enough that the system can rotate in all directions, we decided to align to remove rototranslations before computing the MSD and dMSD. This was done by using `VMD`[8] to align all frames to the last one in every trajectory. Briefly, VMD's alignment plugin [9] iterates over each frame and computes the roto-translation matrix that minimizes the difference between atoms from the current frame and the reference frame using the Kabsch algorithm.

ADDITIONAL EXPERIMENTS AND ANALYSIS

Spatial distribution of minicircles

C. fasciculata has 18 genetically distinct classes of minicircles [1]. Out of these 18 classes we selected a major minicircle sequence (m04) and a minor minicircle sequence (m01) for our analysis. The major class of minicircles constitutes up to 85.2% of the total minicircle population, while the minor class makes up about 10.9% [1].

Using the same dCas9-Qdot 655 labelling approach, and the image anlaysis pipeline discussed in the paper, we quantified the spatial distribution of the major and minor minicircles within the kDNA network. Figures S10 and S11 show the distribution of Qdot655 for the major and minor minicircle classes. The histograms in Figure S11c,e show that the minor minicircles follow a random distribution. The relative frequency graphs in Figure S11d,f show that the major minicircles are slightly enriched at the periphery, with a relative frequency of 1.2 near (but not on) the edge of the kDNA. The major minicircle class are instead depleted at the periphery. The minor minicircles are mostly uniformly distributed, with a relative frequency of close to 1.0 at all the bands.

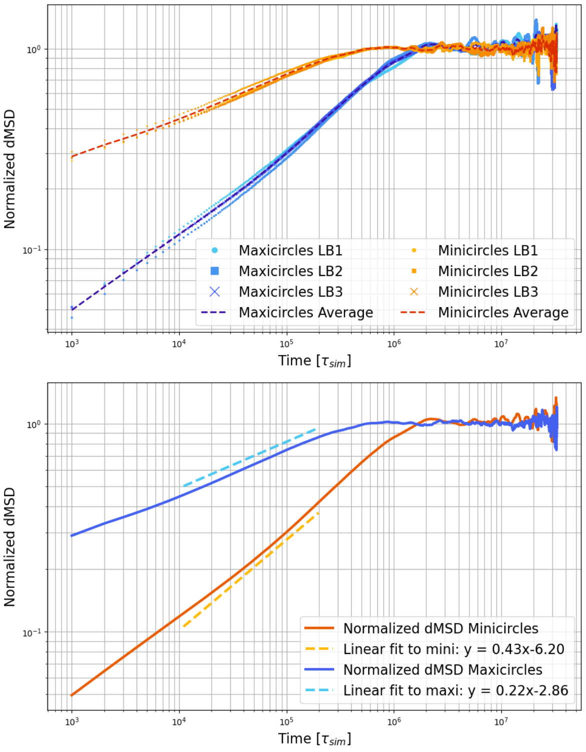

FIG. S9. (ABOVE) Distance Mean Squared Displacement (dMSD) normalized by twice the variance of the vector connecting the two quantum dots, averaged over all the available couples for all the configurations with maxicircles located on the border. The dashed lines represent the average curves. (BELOW) Normalized dMSD of the average curves for both minicircles linked with maxicircles and maxicircles with the corresponding fits.

Dynamics of kDNA

We further analyse the dynamics of the kDNA maxicircles by computing the gyration tensor of the QD and kDNA signals. The gyration tensor is computed as

$$R^t_{\alpha\beta} = \frac{1}{M}\sum_i^M (x^i_\alpha - x^{CM}_\alpha)(x^i_\beta - x^{CM}_\beta) \tag{10}$$

where the sum runs over the "on" pixels in the thresholded image (either QD or kDNA). The value of x^{CM} is the centroid of the on pixels while x^i_α is the α Cartesian component (either x or y) of the i-th pixel. From the tensor, we can compute the gyration radius as the sum of its eigenvalues $R_g^2 = \lambda_1 + \lambda_2$, and the anisotropy from the ratio of the eigenvalues $A = \lambda_1/\lambda_2$.

The values of R_g and anisotropy computed over time from the QD and kDNA signal are shown in Fig. S12. From these values we can compute the autocorrelation, i.e.

$$\xi_I(\Delta t) = \frac{\langle (I(t+\Delta t) - \bar{I})(I(t) - \bar{I})\rangle}{Var(I)} \tag{11}$$

where $Var(I) = \sum_t (I(t) - \bar{I})^2/N$ is the variance and $\bar{I}$ is the mean and the average $\langle \dots \rangle$ is performed over times t.

The autocorrelation of radius of gyration and anisotropy give us a measure of the dynamics of the kDNA and the QDs. When plotted in log-linear scale (See Fig. S12) it is clear that they follow an exponential decay, $\xi_I = A \exp(-t/\tau)$ where τ is some characteristic relaxation time of the kDNA conformation. We typically find values of relaxation times around 5 seconds for both QD and kDNA, which is compatible with previous findings by Yadav and Doyle [10].

Difference between control and PstI-treated kDNA

To quantify the effect of removing maxicircles from the network we used PstI to remove maxircles, gel purified the mini-circle-only network as explained in the text, absorbed onto a poly-L-lysine coated surface and took confocal images. In Fig. S13 we show that the PstI-treated networks are significantly smaller when absorbed on the surface, in line with AFM results published in [1]. We argue that this is due to the network being flatter in bulk and therefore can be absorbed with overall less stretching.

* davide.michieletto@ed.ac.uk

[1] S. Ramakrishnan, Z. Chen, Y. A. G. Fosado, L. Tubiana, W. Vanderlinden, N. J. Savill, A. Schnaufer, and D. Michieletto, PRX Life **2**, 13009 (2024).

[2] P. He, A. J. Katan, L. Tubiana, C. Dekker, and D. Michieletto, Physical Review X **13**, 21010 (2023).

[3] A. A. Hagberg, D. A. Schult, and P. J. Swart, in *Proceedings of the 7th Python in Science Conference*, edited by G. Varoquaux, T. Vaught, and J. Millman (Pasadena, CA USA, 2008) pp. 11 – 15.

[4] J. Chen, C. A. Rauch, J. H. White, P. T. Englund, and N. Cozzarelli, Cell **80**, 61 (1995).

[5] L. Tubiana, G. P. Alexander, A. Barbensi, D. Buck, J. H. Cartwright, M. Chwastyk, M. Cieplak, I. Coluzza, S. Čopar, D. J. Craik, M. Di Stefano, R. Everaers, P. F. Faísca, F. Ferrari, A. Giacometti, D. Goundaroulis, E. Haglund, Y. M. Hou, N. Ilieva, S. E. Jackson, A. Japaridze, N. Kaplan, A. R. Klotz, H. Li, C. N. Likos, E. Locatelli, T. López-León, T. Machon, C. Micheletti, D. Michieletto, A. Niemi, W. Niemyska, S. Niewieczerzal, F. Nitti, E. Orlandini, S. Pasquali, A. P. Perlinska, R. Podgornik, R. Potestio, N. M. Pugno, M. Ravnik, R. Ricca, C. M. Rohwer, A. Rosa, J. Smrek, A. Souslov, A. Stasiak, D. Steer, J. Sułkowska, P. Sułkowski, D. W. L. Sumners, C. Svaneborg, P. Szymczak, T. Tarenzi, R. Travasso, P. Virnau, D. Vlassopoulos, P. Ziherl, and S. Žumer, Physics Reports **1075**, 1 (2024).

[6] L. Tubiana, E. Orlandini, and C. Micheletti, Progress of Theoretical Physics Supplement **191**,

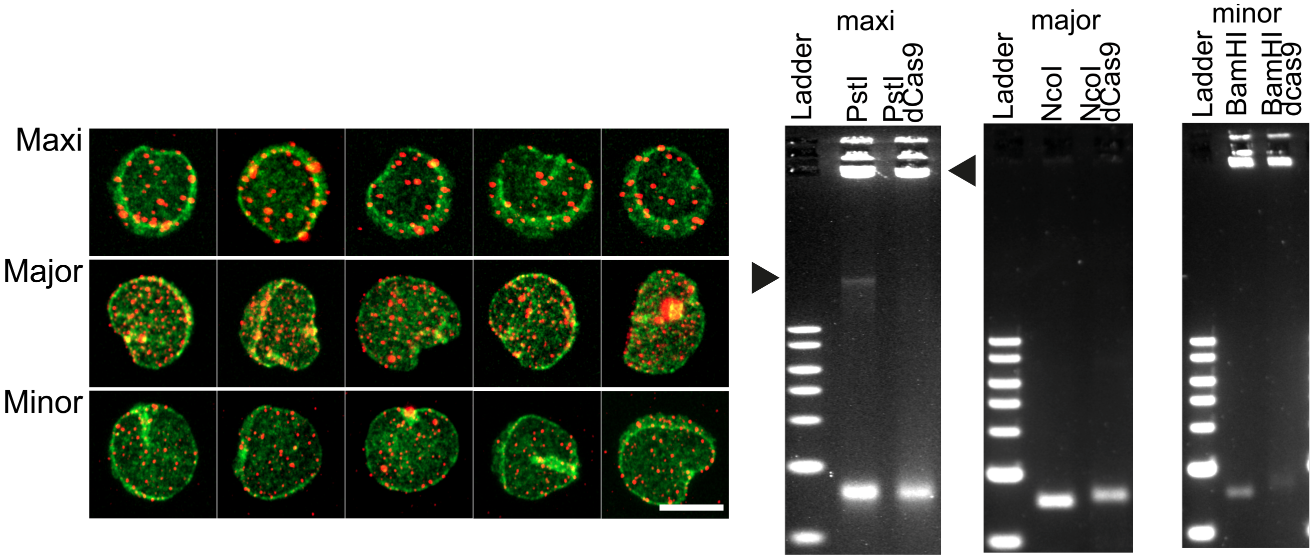

FIG. S10. **QD-labelled kDNA images. left.** Representative images of YOYO-I labelled kDNA (green), Qdot 655 (red) for major and minor minicircle classes and for maxicircles (scale bar at bottom right 5 μm). **right** Gel shift assays demostrating binding of dCas9 to maxicircle and to major and minor minicircle classes.

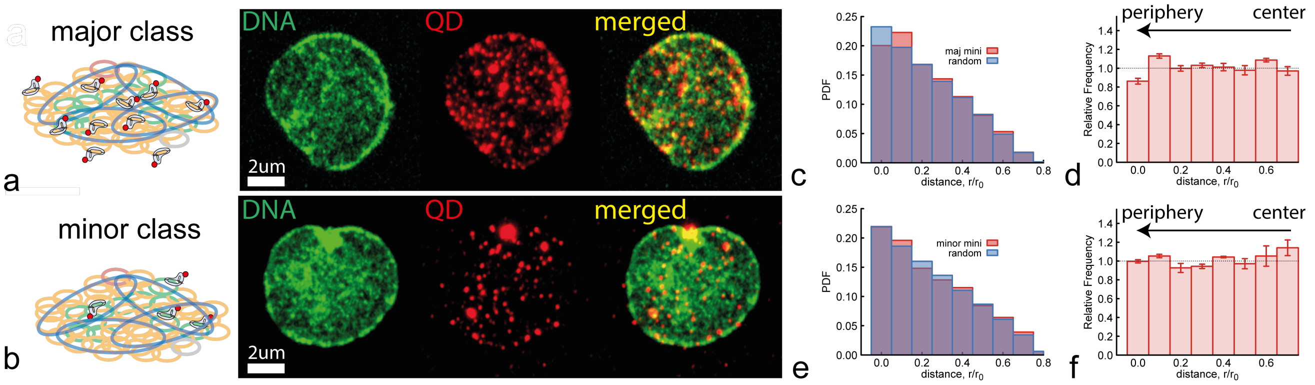

FIG. S11. **Major and minor minicircle classes are mostly uniformly distributed in the kDNA. a-b** Representative images of YOYO-I labelled kDNA (green), Qdot 655 (red) for (a) major and (b) minor minicircle classes. **c,d,e,f** Show (**c,e**) histograms of the distance of Qdot655 from the edge of the network (red) and distances of randomly distributed points (blue) and (**d,f**) normalised frequency of events (observed/random) for the major (top) and minor (bottom) minicircle classes.

192 (2011), https://academic.oup.com/ptps/article-pdf/doi/10.1143/PTPS.191.192/5312306/191-192.pdf.
[7] X. Fang, M. Desbrun, H. Bao, and J. Huang, ACM Trans. Graph. **41** (2022).
[8] J. Eargle, D. Wright, and Z. Luthey-Schulten, Bioinformatics **22**, 504 (2006).
[9] W. Humphrey, A. Dalke, and K. Schulten, Journal of Molecular Graphics **14**, 33 (1996).
[10] I. Yadav, D. Al Sulaiman, and P. S. Doyle, Physical Review Research **5**, 1 (2023).

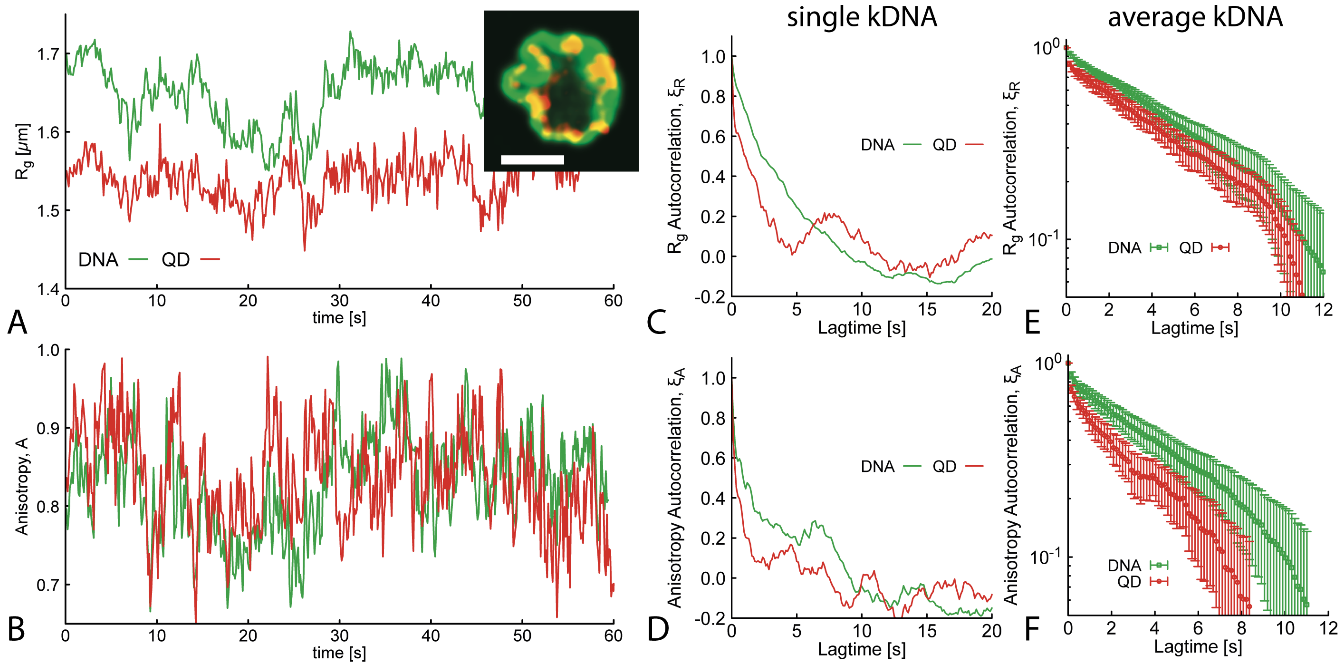

FIG. S12. **A** Radius of gyration computed from the QD signal (red) and the kDNA signal (green). **B** Anistropy computed from the QD signal (red) and the kDNA signal (green). **C** Autocorrelation of the radius of gyration from a single movie. **D** Autocorrelation of the anisotropy from a single movie. **E** Autocorrelation of the radius of gyration averaged over 11 movies plotted in log-linear scale. **F** Autocorrelation of the anisotropy averaged over 11 movies plotted in log-linear scale.

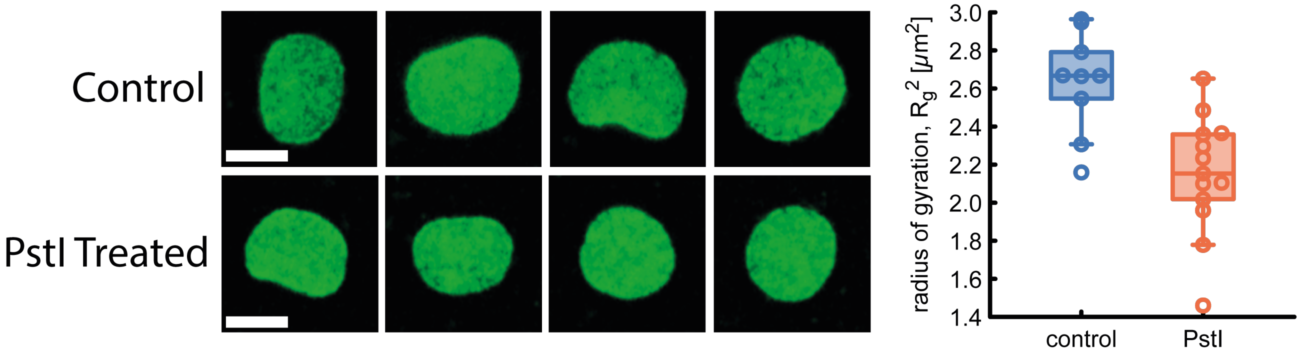

FIG. S13. (Left) Representative images of control (top row) and PstI-treated (bottom row) kDNA networks absorbed on a poly-L-lysine surface. The scale bar is 3.5 μm. (right) Comparison of the radius of gyration of control and PstI-treated kDNA networks. The PstI-treated network displays a systematically smaller size (p-value < 0.001). We argue that this is due to mini-circle-only networks being flatter; they can be fully absorbed with less stretching of the network.
